# Mapping the visual world to the human brain

**DOI:** 10.7554/eLife.75171

**Published:** 2021-12-03

**Authors:** Betina Ip, Holly Bridge

**Affiliations:** 1 Wellcome Centre for Integrative Neuroimaging, Nuffield Department of Clinical Neurosciences, University of Oxford Oxford United Kingdom

**Keywords:** population receptive field, vision, non-human primate, neuroimaging, neurophysiology, Rhesus macaque

## Abstract

The visual maps measured non-invasively in the brain of human and non-human primates reliably reflect the underlying neuronal responses recorded with invasive electrodes.

**Related research article** Klink PC, Chen X, Vanduffel V, Roelfsema P. 2021. Population receptive fields in non-human primates from whole-brain fMRI and large-scale neurophysiology in visual cortex. *eLif*e **10**:e67304. doi: 10.7554/eLife.67304

Vision enables many animals to identify objects and, critically, to locate them in space. To achieve this, a reliable internal map of the world is required. In primates, light enters the eye in an ordered way, creating a map interpreted by the receptors in the retina. The information travels through the optic pathway to the visual brain, where it activates neurons depending on the specific location in the visual field. This ‘receptive field’ is the discrete area of space in the external world that a visual neuron responds to (Figure 1A). Receptive fields can vary in size and include a preference for specific properties, such as orientation or colour (Hubel and Wiesel, 1962). They are essential building blocks of our perception, connecting our internal representation to the external world.

**Figure 1.**
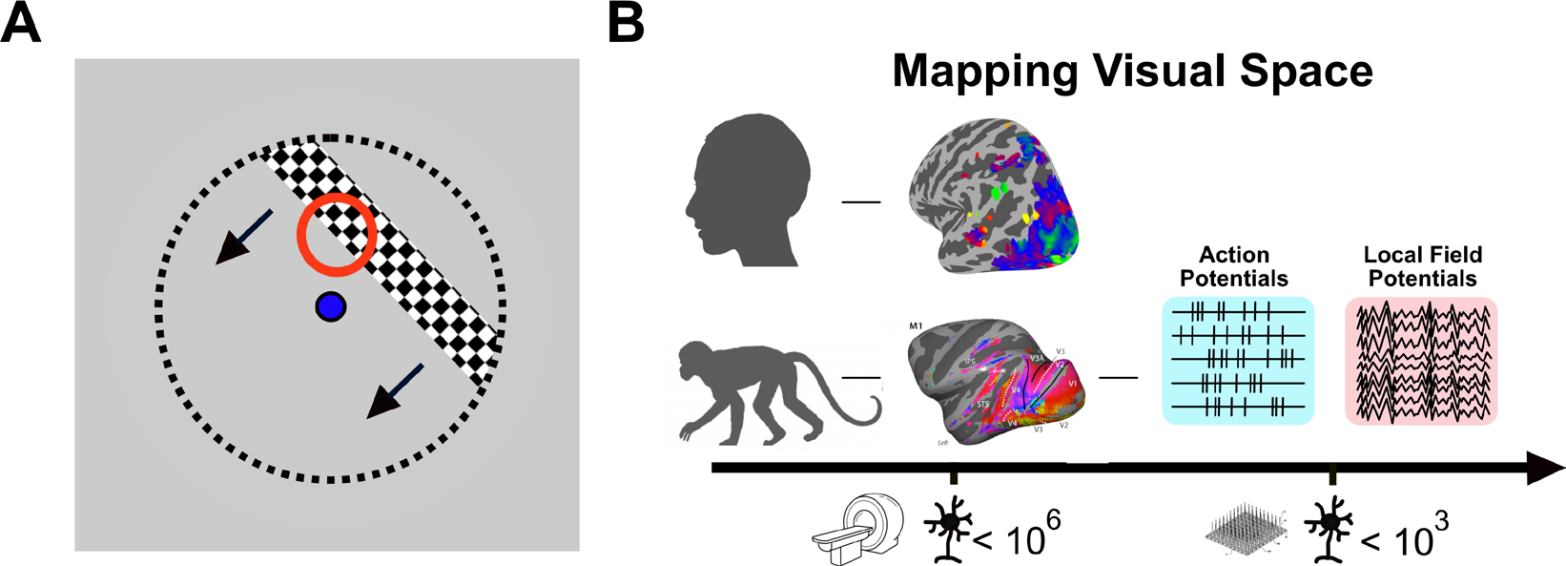
Mapping visual space using invasive and non-invasive methods. (**A**) In experiments studying how neurons respond to visual stimuli, visual space is mapped using a flickering stimulus (black and white bar) that moves repeatedly across a screen in different directions, while the observer looks at the dot in the centre of the screen (blue). As the bar moves through the red circle, some neurons in the visual brain will become active. The red circle represents the ‘receptive field’ of those neurons. The entire visual space is covered with the receptive fields of different neurons. (**B**) In the human brain (top), non-invasive brain imaging is used to study the responses of millions of neurons to visual space by measuring changes in blood flow using fMRI (brain image with colours). In the macaque monkey (bottom), it is possible to directly record neuronal activity during visual stimulation using electrodes (measuring action potentials and local field potentials; right). Since non-invasive imaging and direct neural recordings differ both in spatial scale (scale bar at the bottom) and in the origin of the signal (fMRI measures blood flow and oxygen level as a proxy for increased or decreased activity, while electrodes directly measure the electrical activity of neurons), it is challenging to make comparisons between species and determine the validity of the non-invasive human data.

In animal models, receptive fields can be mapped using microelectrodes to record signals from neurons (Figure 1B). Since this type of invasive approach cannot be used in the human brain, it is necessary to use functional magnetic resonance imaging (fMRI), a technique that indirectly measures the activity of millions of neurons across the brain. The fMRI signal reflects the sum of the activity of all the neurons within a cubic volume called a ‘voxel’; an 8 mm^3^ voxel contains around a million neurons (and fMRI records from thousands of voxels). A human ‘population receptive field’ (pRF) determines the specific region of visual space to which each voxel responds (Dumoulin and Wandell, 2008) and provides a measure comparable to the receptive field of neurons (Figure 1B). However, the fMRI response is an indirect measure of neuronal activity: it records changes in blood flow rather than the electrical activity of neurons. Comparing fMRI data directly to invasive methods is therefore critical to establish whether the fMRI data collected in humans truly reflects the underlying neuronal activity.

Now, in eLife, Christiaan Klink, Xing Chen, Wim Vanduffel and Pieter Roelfsema report that fMRI measurements correspond broadly to direct neuronal recordings (Klink et al., 2021). The team – who are based at the Netherlands Institute for Neuroscience, Amsterdam UMC, KU Leuven Medical School, VU University Amsterdam and Harvard Medical School – first used fMRI to map pRFs across the entire brain in two macaque monkeys. This resulted in clear visual maps of the cortical and subcortical brain regions, comparable to those built out of human data. Using mathematical modelling, Klink et al. determined that responses of neurons were summed non-linearly within each pRF, meaning that different parts of the pRF contributed different amounts to the fMRI output signal. In particular, increasing the stimulus overlap with the pRF did not cause the expected increase in neural response.

Next, Klink et al. analysed multi-unit activity (MUA) data acquired from large, invasive electrode arrays in the visual areas V1 and V4 of two other macaques. These arrays directly measure action potentials from a population of neurons close to each electrode simultaneously. Two analyses were performed: a classical receptive field mapping analysis, using a narrow bar to find the edges of the receptive field (Figure 1A); and a pRF analysis using comparable approaches to the fMRI-based pRF. These yielded similar results, suggesting that the newer pRF technique can be compared to the classical RF technique. Across the two visual areas, receptive fields in V4 were larger than those in V1, reflecting the later stage of processing in V4. The critical result, however, was that the MUA pRFs from both visual areas accurately reflected those derived from the fMRI data.

Klink et al. then analysed pRFs based on local field potentials – the electric potentials in the extracellular space around neurons – recorded simultaneously through all electrodes in the array. While MUA provides information about action potentials in neurons, local field potentials reflect a sum of all electrical activity, both excitatory and inhibitory, including sub-threshold changes not visible in the action potential spike rate (Victor et al., 1994). In many ways, this is more comparable to the fMRI signal, which reflects the oxygen demands of all local activity. Indeed, pRFs derived from local field potentials at specific frequencies that dominate during visual stimulation, also correlated with the fMRI-based pRF, backing previous research suggesting that the change in blood flow correlates best with local field potentials (Logothetis et al., 2001; Berens et al., 2008).

The results of Klink et al. results show that three key features are directly comparable across the three types of pRF measured with invasive and non-invasive approaches. First, all the methods show non-linear summation across the pRF. Second, the size of the pRFs was broadly similar across experiments. Finally, the size of the pRF increases with the distance from where someone is looking. This reflects the high-resolution vision available in the area where someone points their eyes, and the reduction in resolution away from that point. The findings also show that action potentials measured with MUA reflect the fMRI-based pRFs more accurately than the local field potentials, which contrasts with previous studies suggesting that local field potentials better reflect the fMRI signal.

Klink et al. thus show that fMRI and direct neural recordings broadly correspond, indicating that non-invasive brain imaging can reliably reflect receptive fields in the primate brain. Klink et al. exploited the fact that fMRI can measure multiple pRFs across the brain simultaneously to investigate the presence of pRFs outside the visual cortex. It would be valuable to also work in the opposite direction, identifying regions of the brain with interesting or unusual pRF properties for further investigation at a neuronal level. Finally, these spatial maps of the world are present throughout the mammalian visual system, while much of the object identification system is performed in specialised regions. A future challenge will be to apply similar techniques to uncover the mechanisms underlying processing of visual properties such as colour, faces and objects.

## References

[bib1] Berens P, Keliris GA, Ecker AS, Logothetis NK, Tolias AS (2008). Feature selectivity of the gamma-band of the local field potential in primate primary visual cortex. Frontiers in Neuroscience.

[bib2] Dumoulin SO, Wandell BA (2008). Population receptive field estimates in human visual cortex. NeuroImage.

[bib3] Hubel DH, Wiesel TN (1962). Receptive fields, binocular interaction and functional architecture in the cat’s visual cortex. The Journal of Physiology.

[bib4] Klink PC, Chen X, Vanduffel V, Roelfsema P (2021). Population receptive fields in non-human primates from whole-brain fMRI and large-scale neurophysiology in visual cortex. eLife.

[bib5] Logothetis NK, Pauls J, Augath M, Trinath T, Oeltermann A (2001). Neurophysiological investigation of the basis of the fMRI signal. Nature.

[bib6] Victor JD, Purpura K, Katz E, Mao B (1994). Population encoding of spatial frequency, orientation, and color in macaque V1. Journal of Neurophysiology.

